# Blood pressure improvement after stenting in an atherosclerotic renal artery stenosis patient with normal rFFR: A case report

**DOI:** 10.1097/MD.0000000000048846

**Published:** 2026-07-10

**Authors:** Xinyan Wen

**Affiliations:** a Department of Internal Medicine, Peking Union Medical College Hospital, Peking Union Medical College and Chinese Academy of Medical Sciences, Beijing, China; b Department of Cardiology, Peking University First Hospital, Beijing, China.

**Keywords:** atherosclerosis renal artery stenosis, hyperemic transstenotic pressure gradient, renal artery angioplasty, resting pressure gradient, secondary hypertension

## Abstract

**Background:**

There is no consensus on the optimal hemodynamic assessment of renal artery stenosis patients to identify who is suitable for revascularization. Previous studies demonstrated the predictive value of renal fractional flow reserve (rFFR) and hyperemic systolic gradients in blood pressure (BP) improvement after stenting. This report describes BP improvement after stenting in 1 case of atherosclerotic renal artery stenosis with normal rFFR.

**Case presentation:**

A male with the complaint of uncontrollable hypertension was diagnosed with secondary hypertension due to atherosclerotic right renal artery stenosis. The rFFR was 0.96, and the translesion pressure gradient was within normal levels in the rest state but increased to 50 mm Hg in hyperemia. After stent implantation, his BP was normalized.

**Conclusions:**

The case highlights the significance of the assessment of hyperemic systolic gradients in atherosclerotic renal artery stenosis patients with normal rFFR to facilitate decision-making.

## 1. Background

Atherosclerotic renal artery stenosis (ARAS) is the main cause of renovascular hypertension, which increases with age and cardiovascular risk factors.^[1]^ The prevalence depends on different populations, ranging from 2% to 40%.^[2]^ Renal artery stenosis (RAS) can lead to hypertension, renal function impairment, and cardiovascular events. Renal revascularization should be indicated in anatomic significant RAS with several clinical scenarios, including sudden onset of pulmonary edema and continuous kidney function impairment.^[3]^ And some studies demonstrated that a hyperemic systolic gradient (HSG) above 21 mm Hg in patients with unilateral RAS can predict blood pressure (BP) improvement after revascularization. However, there is no consensus on the optimal hemodynamic assessment of RAS. We report BP improvement of an ARAS case after successful revascularization, with normal renal fractional flow reserve (rFFR) and increased hyperemic translesional pressure gradient.

## 2. Case presentation

**Figure 1.**
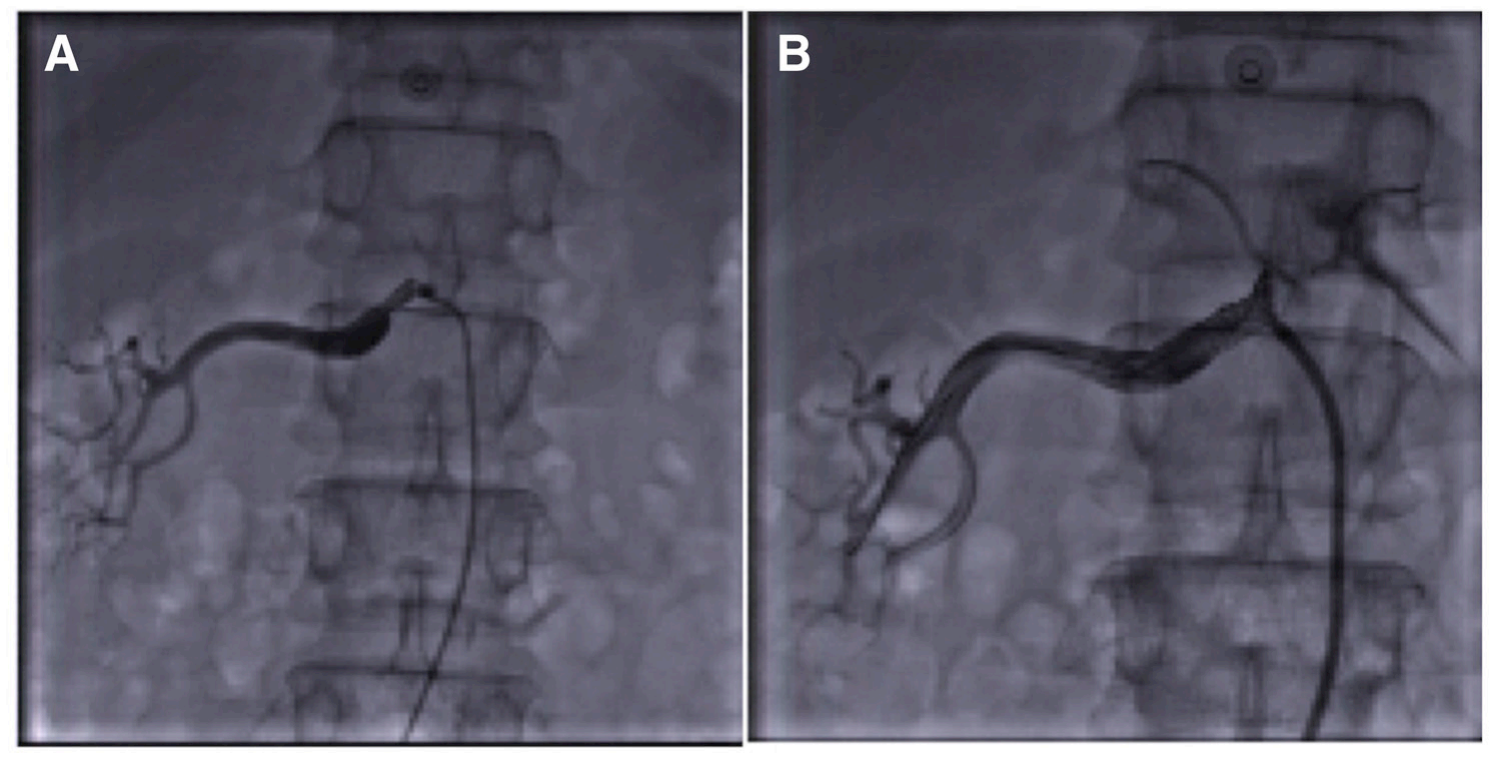
Selective right renal angiography before (A) and after (B) revascularization.

A 52-year-old male was referred to our hospital with the complaint of palpitation and dizziness for 11 months. Several months previously, he presented to a regional hospital for several episodes of sudden onset severe dizziness and palpitation with a BP of 200 mm Hg/110 mm Hg. A plain computed tomography of the head showed no abnormality. Electrocardiogram at that time revealed atrial fibrillation with frequent ventricular rates of 165 bpm. His symptoms resolved, and the electrocardiogram returned to a sinus rhythm with 60 to 70 bpm after administration of antihypertensive regimes and rhythm control therapy. Given the severity and sudden onset of hypertension, a secondary hypertension was suspected. The renal artery ultrasound demonstrated stenosis of 60% in the right renal artery. Kidney ultrasound showed a normal size of the kidney, and a radionuclide renal scintigraphy revealed mild impairment of renal function. Computed tomography of the adrenal gland did not show any suspicious masses. Then a presumptive diagnosis of RAS was made, and he was admitted to our hospital for further investigation. He presented with poorly controlled BP 160 mm Hg/100 mm Hg despite taking multiple antihypertensive regimens (bisoprolol 5 mg once a day and felodipine sustained release tablets 5 mg once a day). He admitted to drinking about 100 g/day of alcohol for the last 30 years and had a 45 pack-years’ smoking history. He denied a family history of hypertension or cardiovascular disease. Apart from high BP, the result of her physical examination was unremarkable. Laboratory examination at admission showed normal plasma creatinine. Biochemical screening for secondary hypertension found serum thyroid-stimulating hormone, the circadian rhythm of cortisol, catecholamines, metanephrines, and aldosterone to renin ratio within reference levels and negative autoimmune screening. Renal artery computed tomography angiography visualized a stenotic right renal artery, confirming the suspected RAS. With consideration of clinical characteristics suggestive of atherosclerosis, including old age, smoking, and drinking history, he was tentatively diagnosed as having hypertension secondary to ARAS. For diagnosis confirmation and further intervention, renal angiography was performed (Fig. 1). After insertion of a 6F guiding catheter through the right femoral artery, 60% right renal artery ostial stenosis was found in the selected right renal angiography. A 0.014 inch pressure guidewire (Pressure Guidewire, Abbott), which was advanced across the lesion after calibration, was used to measure the resting pressure gradient. The value of rFFR (defined as the ratio of mean arterial pressure distal to the stenosis [Pd] to the mean aortic pressure measured from the guiding catheter [Pa], as follows: rFFR = Pd/Pa) was 0.96, and the mean artery pressure gradient at rest was below 10 mm Hg before the revascularization. Hyperemia was induced with intrarenal bolus injection of 50 µg kg^−1^ dopamine. And the hyperemic transstenotic pressure gradient was 50 mm Hg, which indicates hemodynamic significance of the stenosis. Then he was finally diagnosed with secondary hypertension secondary to ARAS with hemodynamic significance. In view of poorly controlled hypertension, mild impairment of right kidney function, and the hope of the patient, right renal artery angioplasty with stent was performed. The lesion of the right renal artery was dilated with a 5.0 mm × 12 mm balloon at 12 atm, and a balloon-expandable stent (7.0 mm × 18 mm; RX Herculink elite, Abbott) at 8 atm was deployed successfully. The final angiograms showed successful restoration of the right renal artery blood flow, with no residual translesion pressure gradient (FFR 1.00, pressure gradient 5 mm Hg after revascularization). Perclose ProGlide (Abbott Vascular) was used to close the femoral access site. After the procedure, dual antiplatelet therapy with aspirin and ticagrelor was initiated to prevent stent thrombosis, and his BP decreased to 130 to 140/80 to 90 mm Hg with bisoprolol 5 mg once a day and felodipine sustained-release tablets 5 mg once a day. The patient discontinued felodipine sustained-release tablets and ticagrelor by himself after 6 months without any adverse event. And his BP was normalized with bisoprolol 5 mg daily to improve the symptom of palpitation. A smoking cessation education was also provided for the patient.

## 3. Discussion and conclusions

This case highlights the significance of hyperemic pressure gradient in the decision-making for further intervention in ARAS patients with normal rFFR.

Previous randomized controlled trials showed no additional benefit from percutaneous transluminal renal angioplasty (PTRA) compared with guideline-directed medical treatment for hypertensive patients with ARAS in renal and cardiovascular outcomes.^[4–7]^ Consequently, the 2017 American Heart Association guideline weakly recommended revascularization in ARAS for whom medical management has failed (IIb, level of evidence C).^[8]^ However, there are significant flaws in these studies’ design, including small size, patients selected mainly based on angiographic parameters, and few participants with severe stenosis, defined as >80% blockage of an artery. PTRA could significantly increase the GFR of a stenotic kidney and improve ambulatory BP in ARAS patients despite total renal function and office BP remaining unchanged.^[9]^ PTRA with stenting could reduce the incidence of restenosis and is the treatment of choice for renovascular hypertension patients with ARAS.^[10]^

Previous studies demonstrated that the hyperemic translesional gradient or rFFR could predict patients’ BP response after stenting. HSG above 20 mm Hg has been proposed to identify functionally significant RAS, since pressure gradients at rest may underestimate the significance of the stenosis due to the existence of renal parenchymal resistance. Leesar et al^[11]^ and Jones et al^[12]^ reported significant hypertension improvement in hypertensive patients with unilateral RAS who had an HSG >20 mm Hg. Mangiacapra et al^[13]^ reported that dopamine-induced mean pressure gradient ≥20 mm Hg provided the highest predictive value on pressure improvement in patients with renovascular hypertension after renal artery stenting. Prior study also found that RAS with rFFR below 0.90 was significantly associated with renin release, which can be considered as hemodynamically significant stenosis.^[14]^ Mitchell et al^[15]^ proved that rFFR was an independent predictor of BP improvement. And the result of the fractional flow reserve to determine the Appropriateness of percutaneous renal artery intervention in atherosclerotic renovascular hypertension patients (FAIR) study, which involved 101 patients with ARAS and hypertension, showed that FFR <0.8 is the optimal cutoff predicting BP reduction after stenting.^[16]^

Hemodynamic assessment plays an important role in identifying appropriate patients who are suitable for revascularization. However, the optimal hemodynamic assessment remains inconclusive. Our case indicated that HSG should be obtained for decision-making for revascularization in ARAS patients with normal rFFR. Further study is warranted to explore the optimal hemodynamic assessment method of RAS.

## 4. Limitations

The epidemic of COVID-19 and long-distance make details on follow-up examinations at regular intervals unavailable. The use of antithrombosis therapy should be further assessed to prevent the risk of thrombosis secondary to atrial fibrillation.

## Author contributions

**Conceptualization:** Xinyan Wen.

**Resources:** Xinyan Wen.

**Supervision:** Xinyan Wen.

**Validation:** Xinyan Wen.

**Writing – original draft:** Xinyan Wen.

**Writing – review & editing:** Xinyan Wen.
